# Towards Real-Time Heartbeat Classification: Evaluation of Nonlinear Morphological Features and Voting Method

**DOI:** 10.3390/s19235079

**Published:** 2019-11-21

**Authors:** Rajesh N V P S Kandala, Ravindra Dhuli, Paweł Pławiak, Ganesh R. Naik, Hossein Moeinzadeh, Gaetano D. Gargiulo, Suryanarayana Gunnam

**Affiliations:** 1 Department of ECE, GVPCE (A), Visakhapatnam 530048, India; kandala.rajesh2014@gmail.com; 2 Department of ECE, VIT University, Andhra Pradesh 522237, India; ravindradhuli@gmail.com; 3 Department of Information and Communications Technology, Faculty of Computer Science and Telecommunications, Cracow University of Technology, Warsaw 24 st., F-3, 31-155 Krakow, Poland; 4 Institute of Theoretical and Applied Informatics, Polish Academy of Sciences, Bałtycka 5, 44-100 Gliwice, Poland; 5 The MARCS Institute, Western Sydney University, Milperra, NSW 2214, Australia; ganesh.naik@westernsydney.edu.au (G.R.N.); h.moeinzadeh@westernsydney.edu.au (H.M.); g.gargiulo@westernsydney.edu.au (G.D.G.); 6 Department of Electrical Engineering and Information Technology (DIETI), “Federico II” The University of Naples, 80100 Naples, Italy; 7 School of Engineering at Western Sydney University, Penrith, NSW 2747, Australia; 8 Institute of Image Processing and Pattern Recognition, Shanghai Jiao Tong University, Shanghai 200240, China; surya_gunnam@yahoo.co.in

**Keywords:** electrocardiogram signal, nonlinear features, improved complete ensemble empirical mode decomposition, inter-patient scheme, voting, classification, FPGA

## Abstract

Abnormal heart rhythms are one of the significant health concerns worldwide. The current state-of-the-art to recognize and classify abnormal heartbeats is manually performed by visual inspection by an expert practitioner. This is not just a tedious task; it is also error prone and, because it is performed, post-recordings may add unnecessary delay to the care. The real key to the fight to cardiac diseases is real-time detection that triggers prompt action. The biggest hurdle to real-time detection is represented by the rare occurrences of abnormal heartbeats and even more are some rare typologies that are not fully represented in signal datasets; the latter is what makes it difficult for doctors and algorithms to recognize them. This work presents an automated heartbeat classification based on nonlinear morphological features and a voting scheme suitable for rare heartbeat morphologies. Although the algorithm is designed and tested on a computer, it is intended ultimately to run on a portable i.e., field-programmable gate array (FPGA) devices. Our algorithm tested on Massachusetts Institute of Technology- Beth Israel Hospital(MIT-BIH) database as per Association for the Advancement of Medical Instrumentation(AAMI) recommendations. The simulation results show the superiority of the proposed method, especially in predicting minority groups: the fusion and unknown classes with 90.4% and 100%.

## 1. Introduction

### 1.1. Aim of the Work

Nowadays, mortality rates are increasing due to noncommunicable diseases (NCDs) over infectious diseases. Annually about 70% of deaths are because of the NCDs worldwide. As per the World Health Organization (WHO) report, cardiovascular diseases (CVDs) are the primary cause of death among other NCDs [1]. The effect of CVDs is more in low and middle-income countries. The report demonstrates that this impact will continue further. This alarming scenario influences not only the health perspective, but also the socio-economic advancement of the country. Therefore, the need for adequate diagnosis and treatment for NCDs, especially for CVDs, is highly essential. This situation demands advancements in healthcare technology.

Cardiac arrhythmias are one of the significant sources of CVDs. All arrhythmias may not be fatal, but some need immediate treatment for the patient to survive. Arrhythmias may occur owing to erratic electrical impulse conduction or formation in the heart. Electrocardiograms (ECGs) are an essential tool to study the electrical activity of the heart. ECG discloses any variations in the heartbeat pattern. Clinicians have to explore the longer duration ECG records in the diagnosis process. However, this manual examination is tiresome because of low amplitude and subtle variations in the ECG [2]. Hence, computer-aided diagnosis (CAD) helps clinicians remarkably. CAD-based heartbeat classification is a significant task before the arrhythmia recognition.

### 1.2. State-of-the-Art

If we look at the literature on heartbeat classification, it can be observed that the Massachusetts Institute of Technology-Beth Israel hospital (MIT-BIH) arrhythmia database [3] is the majority choice. The essential literature of heartbeat classification using this database can be categorized into two types based on the assessment process, viz., class-oriented and subject-oriented. The majority among them are class-oriented based works [4,5,6,7,8,9,10,11,12,13,14,15,16,17,18,19,20,21,22].

In the class-oriented approach, from the 16 types of beats including the normal ones in the MIT database, a part or an entire collection of beats are preferred for classification. In [4], 17 types of heartbeats including normal and pacemaker are classified using the features based on various power spectrum density methods. Later, a novel genetic algorithm is used to identify the optimum features to enhance the classification process. Finally, these selected features are fed to the various standard machine learning algorithms. In [19], 13 types of heartbeats are classified using the combination of higher-order statistics (HOS) of the ECG and Hermite basis representation features using a support vector machine (SVM) classifier. In [18], six types of heartbeats are classified by using a local fractal dimension based nearest neighbor classifier. In [21], seven types of heartbeats are classified using gray relational analysis. In [20], Ye et al. designed a heartbeat classification algorithm using dynamic and morphological ECG features. For the morphological feature extraction process, the combination of wavelet transform and the dimensionality reduction technique, namely independent component analysis (ICA), is implemented on the heartbeats. R–R intervals are used as dynamic features. These features are then fed to SVM for classifying 16 types of heartbeats. In [8], a novel genetic ensemble of classifiers machine learning method is proposed. A new genetic training coupled with genetic optimization is used to classify 17 types of heartbeats. In [17], statistical and nonlinear features are derived from the modes obtained from the empirical mode decomposition (EMD) algorithm. Later, these features are provided to one-against-one SVM for classifying five types of heartbeats. In [22], ventricular extra systole or ectopic beats are recognized with the help of morphology matching, R–R intervals, and clustering algorithms. In [6], 17 types of EG beats are classified using hexadecimal local patterns claculated from wavelet sub-bands. In [7], five primary types of heartbeats are classified using ensemble empirical mode decomposition (EEMD) based features subjected to sequential minimal optimization-SVM (SMO-SVM). Besides, Neural networks plays a crucial role in biological signal analysis [23]. Recently, deep learning-based class-oriented schemes come into the picture. Deep learning techniques are a part of machine learning techniques implemented based on more hidden neural networks. In [9,10] these works, 17 types of heartbeats are classified using 1D-CNN and a novel 3-layer deep genetic ensemble of classifiers.

In the subject-oriented approach, the entire MIT-BIH database is subdivided into five groups of heartbeats according to the American National Standards Institute/Advancement of Medical Instrumentation (ANSI/AAMI) EC57:1998 standard. The list of these groups is non-ectopic (N), supraventricular ectopic (S), ventricular ectopic (V), fusion (F), and unknown (Q). Again, two strategies are observed for classifying these distinct groups: intra-patient and inter-patient schemes. The fundamental disagreement between these two strategies is the separation of training and testing datasets. Intra-patient scheme based methods are widely explored in the literature [24,25,26,27,28,29,30]. However, these approaches have less impact in real-time scenarios. Because, in real-time applications, an unknown subject that usually undergoes the testing will be foreign to the constructed model. Thus, the model has to be adequate to capture the inter-individual variations among the ECG. While designing the intra-patient based model, there might be a chance of having common subject information in both training and testing. To mitigate such an issue, De Chazal et al. [31] introduced an inter-patient scheme based heartbeat classification. Here, the overall MIT-BIH database is separated into two groups. One group is assigned to training, and the other one is for testing by ensuring that there is no similar subject data in both groups.

The advantage of the aforementioned computer-aided expert systems can be exploited only after developing real-time systems. In literature, in recent years, some of the field-programmable gate array (FPGA) based ECG signal analysis systems are implemented. In [32], an FPGA based heartbeat classification system is developed using the least-squares linear-phase finite impulse response filter and feed-forward neural network. In [33], three types of common arrhythmia beats, namely, premature ventricular contraction, ventricular fibrillation, and heart block beat along with normal beats, are classified using a real-time FPGA implementation. In [34], an intra-patient scheme based on arrhythmia classification is implemented in the FPGA system. However, most of the successful FPGA implemented systems are followed by an intra-patient scheme. Very few methods are developed in real-time systems based on inter-patient schemes [35]. However, still, these systems failed in detecting rare abnormal beats accurately. Hence, there is a need for developing a new expert system that can succeed in identifying rare heartbeats.

### 1.3. Contribution

In this paper, we presented an efficient inter-patient heartbeat classification algorithm. For any pattern recognition process, identifying an appropriate set of features and classifier is highly significant. From [36], it is noticeable that ECG is a non-stationary, non-Gaussian signal derived from nonlinear systems. Hence, we employed a decomposition method, namely improved complete ensemble empirical mode decomposition (ICEEMD) to obtain features from the ECG beats. This technique is capable of disclosing the implicit information lying in the ECG. Later, different nonlinear measures like entropies and HOS are determined from the modes obtained after ICEEMD. These measures will serve as features for proper discrimination of the heartbeat groups. The fundamental difficulty in processing these groups is the class imbalance. Here, a significant fraction of the heartbeats is non-ectopic. Hence, the results may be biased toward the majority group, which is undesirable. Therefore, to alleviate such an issue, we followed an algorithmic level approach. To achieve this, we employed a majority voting scheme based classification. It is a type of ensemble classification. The advantage of ensemble classification is that it can reduce both variance and bias. In this work, we used different combinations of classifiers, namely, naïve Bayes, linear, and quadratic discriminant functions, J48, and consolidated J48 classifiers for majority voting.

The rest of the paper is ordered as follows: the ECG data set, training, and testing data division of AAMI labeling, experimental details and theoretical background of the methodology are presented in Section 2. Section 3 presents the simulation results of the proposed method. The comparison with existing works, limitations, and future directions are presented in Section 4. The conclusions of the work are presented in Section 5.

## 2. Methods

**Figure 1:**
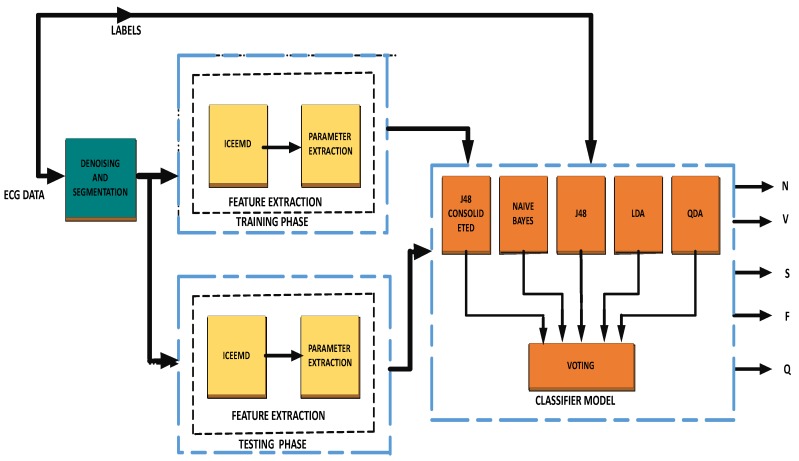
Block diagram of the proposed methodology.

The block diagram of the proposed method is illustrated in Figure 1. The methodology consists of three stages including pre-processing, feature extraction on training and testing data, and a classification model for evaluation. In this section, the database used and the theoretical background of the used techniques are discussed.

### 2.1. Database

The proposed method is examined using the MIT-BIH arrhythmia database. MIT-BIH is a standard database widely explored for arrhythmia classification. It comprises of Holter monitoring records from several male and female patients. Each record duration is 30 minutes, sampled at 360 Hz. The records consist of both normal and abnormal beats of 15 types.

The annotation files available in the database are obtained from the chart recordings recognized by the experts. This file describes the ‘R’ peak locations and the labeling of normal and abnormal beats. Based on the recommendations of AAMI, class-labeling was assigned for discriminating various heartbeat groups.

#### AAMI Class Labeling Recommendations

According to the ANSI/AAMI EC57:1998 standard, within the annotation files, beat labels are divided into five groups, namely, N, S, V, F, and Q based on the physiological origin of the beats. Here, the mainly N group consists of normal and bundle branch block beats. S and V groups consist of ectopic beats, originated above and below Atrio Ventricular (A–V) junction of the heart, respectively. The F group consists of the combination of ventricular and normal beats. Unclassifiable beats are placed in the Q group. According to [31], the total number of available heartbeats are divided into training for modeling and testing for evaluation. Details of the number of heartbeats utilized for this work are presented in Table 1.

### 2.2. Pre-Processing

Pre-processing is an initial step in any data processing systems. Raw ECG signals will inherently have some artifacts. These may occur due to instrumental noise (power line interference), a physiological signal disturbance (muscular movements), or the environment where the experiment takes place. These artifacts are undesirable and diminish significant features in the ECG. Therefore, to attenuate the effect of this noise, we perform denoising as one of the pre-processing steps. For this, we used a filtering routine proposed by [37] with minimal modification. This operation comprises the following:

1. Mean separation from the noisy ECG,
2. Moving average filter of order five,
3. High-pass filter with cut-off frequency 1 Hz (for baseline wander suppression),
4. Low pass Butter worth filter with cut-off frequency 45 Hz (To suppress any left out high-frequency noise).

We need individual heartbeats from the long-term ECG recording for heartbeat classification. We perform a segmentation process after denoising. In the segmentation process, the annotation chart records with ‘R’ peak locations are utilized. From the annotation file, it is observed that there are a lot of variations among R-peak positions time-to-time. The difference between the R-peak positions is dynamic. Hence, we applied a window of length 300 samples on ECG signal to obtain an ECG segment that covers the QRS complex which is an important epoch in the ECG. Our segmentation process retains other important epochs like P and T waves, unlike centered R-peak distribution segmentation methods.

### 2.3. Feature Extraction

Feature extraction has a critical role in heartbeat classification. A feature provides crucial information about a signal and facilitates better discrimination of classes. From [36], it is evident that ECG is a non-stationary signal stemming from a nonlinear system. Hence, exploration of ECG with nonlinear methods can improve the performance of a model since they extract subtle information lying in ECG. Therefore, in the feature extraction stage, initially, we perform ICEEMD on ECG segments to get intrinsic mode functions (IMFs). Later, entropy and higher-order cumulants are extracted from the selected modes. In this section, the techniques employed and their support in the methodology development are briefly discussed.

#### 2.3.1. ICEEMD

The EMD decomposes a given signal in a full data-dependent approach by exploiting the local characteristics. However, EMD is limited by “mode-mixing’’ problem while analyzing the real data [38]. Therefore, some noise-assisted data analysis methods can provide a solution. Here, noise is added in a controlled manner for developing new extrema. Thus, the local mean is limited to that of the original version where extrema are generated. A few among these noise assisted methods are EEMD [39] and CEEMD [40]. Among these methods, CEEMD provides a better solution to the mode-mixing problem. However, CEEMD has some limitations:

(i) Some residual can be present in the modes.
(ii) During the initial decomposition stages, information may appear “late” with undesired modes, when it is compared to EEMD.

To address these issues, Colominas et al. [41] introduced a new noise aided adaptive data analysis method called ICEEMD. The mathematical details of the ICEEMD are given below [41].

Notation used in algorithm: $E_{l}\left(.\right)=l^{th}$ EMD mode, M(.)= local mean of the signal, <.>= averaging operator, $w^{\left(j\right)}=$ realization of white Gaussian noise with zero mean and unit variance and *x* = input signal.

The algorithm steps:

1. Compute the local means of *J* realizations $x^{\left(j\right)}=x+\beta_{0}E_{1}\left(w^{\left(j\right)}\right),j=1,2,\ldots ,J$ using EMD, to obtain first residue $r_{1}=<M\left(x^{\left(j\right)}\right)>$.
2. At the first stage (l=1), compute the first IMF:
  (1) $$C_{1}=x-r_{1}.$$
3. For l=2,…,L, calculate $r_{l}$ as
  (2) $$r_{l}=<M(r_{l-1}+\beta_{l-1}E_{l}\left(w^{\left(j\right)}\right)>.$$
4. Calculate the $l^{th}$ mode as
  (3) $$C_{l}=r_{l-1}-r_{l}.$$
5. Go to step 3 for next *l*

Here, $\beta_{l}=\epsilon_{0}\sigma_{\left(r_{l}\right)}$ is used to obtain the desired SNR at each stage. We choose $\epsilon_{0}=0.2$. The resultant IMFs provide significant underlying features of the ECG signal. The ICEEMD is a beneficial tool used for analyzing non-stationary signals originating from nonlinear systems such as bio-signals. The main advantage of ICEEMD is: avoiding the spurious modes and reducing the amount of noise in the mode patterns. Thus, the decomposed IMFs capture the morphology of the signal. Later, entropy and statistical measures are calculated from the first six modes of each ECG segment.

#### 2.3.2. Entropy Measures

Entropy measures the uncertainty in a given data. It is often used in signal processing and pattern recognition applications [42]. A high value of entropy maps to higher uncertainty (or) unpredictability. Entropy yields useful information for analyzing non-stationary signals [43]. In this work, we calculated Shannon [44], log energy, and norm entropies [45]. The entropy *E* must be an additive cost function such that E(0)=0 and

$$E\left(s\right)=\sum_{i}E\left(s_{i}\right),$$

where *s* is the probability of the given signal and *i* represents one of the discrete states. Various entropies are defined below:

- **Shannon Entropy:**
  (4) $$E_{\mathrm{Shannon}}\left(s\right)=\sum_{i}s_{i}^{2}\log\left(s_{i}^{2}\right),$$
- **log Energy Entropy:**
  (5) $$E_{\log\;\mathrm{energy}}\left(s\right)=\sum_{i}\log\left(s_{i}^{2}\right),$$
  with the convention log(0)=0.
- **norm Entropy:** The $l^{p}$ norm entropy with 1≤p is defined as
  (6) $$E_{\mathrm{norm}}\left(s\right)=\sum_{i}|\left(s_{i}^{p}\right){\left|=||s|\right|}_{p}^{p}.$$

#### 2.3.3. HOS

HOS provides a meaningful measure for analyzing non-stationary signals originating from nonlinear systems [46,47]. HOS represents the deviation from Gaussianity and can provide useful information from the non-Gaussian nature of ECG signals. In our work, we utilized second, third, and fourth-order cumulants as HOS. The mathematical details of the HOS can be found in [48].

We construct a feature vector of size 36×1 for each heartbeat (6 features × 6 modes = 36). Later, the training feature set is fed to a classifier for building a model, and that model is evaluated using a testing set.

### 2.4. Voting Scheme

The final goal of machine learning is to get better-generalized performance. We come across a question “which learning algorithm or classifier is preferred over the other ?’’. According to a “No free launch theorem’’ [49], there is no precise answer to this. One algorithm fits or performs well for a set of training and testing data and may fail for another. The learning algorithm overall performance depends on the prior information, distribution of data, amount of training data, and some cost functions. The performance generalization depends on the bias and variance errors. Always, there will be a trade-off between bias and variance. Ensemble classifiers form a better choice, to improve the performance generalization by reducing bias and variance. Combining several classifiers for the final decision is called an ensemble classification or mixture-of-experts model or modular classification.

The primary motivation behind the classifier ensemble is improving the classification performance using the complementary information offered by various classifiers. Kittler et al. [50] developed a scheme for combining classifiers using voting based on a set of rules: min-rule, max-rule, product-rule, sum-rule, and median-rule. From our experiments, we preferred product rule which outperforms others.

**Mathematical Framework:** Consider a pattern recognition model where a pattern y is to be assigned with one of the *m* possible classes $\left(\omega_{1},\omega_{2},\ldots \ldots ,\omega_{m}\right)$. Say there are *R* number of classifiers used for combining. Let us assume that each classifier possesses a different representation of measurement vector $x_{i},i=1,2,\ldots ,R$.

The density function for each class $\omega_{k}$ in the measurement space is $p(x_{i}|\omega_{k})$ and the prior probability is $P(\omega_{k})$. We assume that the models are mutually exclusive.

From the Bayesian framework, y is assigned to the class $\omega_{j}$ having a maximum posterior probability out of $\omega_{k}$ classes:

(7) $$\begin{array}{c} \mathrm{assign}\;y\to \omega_{j}\;\mathrm{if}\;P\left(\omega_{j}|x_{1},x_{2},.\ldots .,x_{R}\right)=\max_{k}P\left(\omega_{k}|x_{1},x_{2},.\ldots .,x_{R}\right). \end{array}$$

Rewriting the posterior probability $P(\omega_{k}|x_{1},x_{2},.\ldots .,x_{R})$ using the Bayes theorem:

(8) $$P\left(\omega_{k}/x_{1},x_{2},.\ldots .,x_{R}\right)=\frac{p\left(x_{1},x_{2},.\ldots .,x_{R}|\omega_{k}\right)P\left(\omega_{k}\right)}{P(x_{1},x_{2},.\ldots .,x_{R})}.$$

Here, $P(x_{1},x_{2},.\ldots .,x_{R})$ can be expressed in terms of conditional measurement distribution as

(9) $$p\left(x_{1},x_{2},.\ldots .,x_{R}\right)=\sum_{j=1}^{m}p\left(x_{1},x_{2},.\ldots .,x_{R}|\omega_{j}\right)P\left(\omega_{j}\right).$$

**Product Rule:**$p(x_{1},x_{2},.\ldots .,x_{R}|\omega_{j})$ represents the joint probability distribution of the measurements computed by the classifiers. Assuming that these representations are statistically independent, we can rewrite the joint probability distribution as

(10) $$p\left(x_{1},x_{2},.\ldots .,x_{R}|\omega_{k}\right)=\prod_{i=1}^{R}p\left(x_{i}|\omega_{k}\right).$$

Based on $p(x_{i}|\omega_{k})$, the measurement process model for $i^{th}$ representation is developed. Substituting Equation (10) and Equation (9) into Equation (8)

(11) $$P\left(\omega_{k}|x_{1},x_{2},.\ldots .,x_{R}\right)=\frac{P\left(\omega_{k}\right)\prod_{i=1}^{R}p\left(x_{i}|\omega_{k}\right)}{\sum_{j=1}^{m}p\left(\omega_{j}\right)\prod_{i=1}^{R}p\left(x_{i}|\omega_{j}\right)}$$

and using Equation (11) in Equation (7), we obtain the decision rule

(12) $$\mathrm{assign}\;y\to \omega_{j}\;\mathrm{if}\;P\left(\omega_{j}\right)\prod_{i=1}^{R}p\left(x_{i}|\omega_{j}\right)=\max_{k=1}^{n}P\left(\omega_{k}\right)\prod_{i=1}^{R}p\left(x_{i}|\omega_{k}\right).$$

Rewriting in terms of the posterior probabilities obtained from the respective learning algorithms,

(13) $$\mathrm{assign}\;y\to \omega_{j}\;\mathrm{if}\;\frac{1}{P^{\left(R-1\right)}\left(\omega_{j}\right)}\prod_{i=1}^{R}p\left(\omega_{j}|x_{i}\right)=\max_{k=1}^{n}\frac{1}{P^{\left(R-1\right)}\left(\omega_{k}\right)}\prod_{i=1}^{R}p\left(\omega_{k}|x_{i}\right).$$

Equation (13) represents the likelihood decision rule obtained after combining the posterior probabilities generated by different classifiers using the product rule.

In this work, we used five different classifiers for ensembling using a voting scheme to enhance the performance of the system: naïve Bayes [51], linear and quadratic discriminant functions [52], J48 [53], and J48 consolidated classifiers [54]. A brief description of these classifiers is given below.

**Naïve Bayes Classifier:** It is a probability-based learning algorithm developed on the Bayesian framework. According to Bayes theorem, an unknown y is categorized into the one among the *R* classes, with high posteriori probability:

(14) $$y\to \omega_{k}\;\mathrm{if}\;\arg\;\max_{\omega_{k}\in \omega }P\left(\omega |y\right)P\left(y\right),$$

where $\omega =\left\{\omega_{1},\omega_{2},\ldots .,\omega_{R}\right\}$ is a vector of *R* classes.

Naïve Bayes is a modified version of Bayes classifier, based on the assumption that the features in an unknown example vector are independent. Therefore, posteriori probability can be written as

(15) $$P\left(\omega |y\right)=P\left(y|\omega \right)P\left(\omega \right)=P\left(y_{1},y_{2},\ldots ,y_{m}|\omega \right)=P\left(y_{1}|\omega \right)P\left(y_{2}|\omega \right)\ldots \ldots P\left(y_{m}|\omega \right)P\left(\omega \right).$$

Hence, Equation (14) can be modified as

(16) $$y\leftarrow \arg\;\max_{\omega_{k}}P\left(\omega =\omega_{k}\right)\prod_{i}P\left(y_{i}|\omega =\omega_{k}\right).$$

With this final rule, the naïve Bayes classifier operates. The parameters used for the Naïve Bayes Classifier is given in Table 2.

In general, the naïve Bayes classifier assumes that the given features follow the normal distribution. In Table 2, use the Kernel Estimator parameter set to false to follow this assumption. Supervised discretization converts a specific range of attribute values to binary values. Here, the term supervised is coined because the class information of the training instances is used for discretization. However, this process is possible only when the class labels are nominal. The advantage of supervised discretization in naïve Bayes classifier is present in [55].

**Linear and Quadratic Discriminant Analysis Based Classifiers:** The approach of discriminant analysis is to derive a decision boundary or a discriminant function based on the linear combinations of features that best separate the given classes. The assumption made is: examples from different categories follow Gaussian distribution. For instance, the discrimination function for two-class problems based on Bayes theory can be written as

(17) $${\left(y-\mu_{1}\right)}^{T}\Sigma_{1}^{-1}\left(y-\mu_{1}\right)+\ln\left|\Sigma_{1}\right|-{\left(y-\mu_{2}\right)}^{T}\Sigma_{2}^{-1}\left(y-\mu_{2}\right)>T,$$

where $\mu_{1},\mu_{2}$ are the mean vectors of class1 and class 2, $\Sigma_{1},\Sigma_{2}$ are the covariance matrices of class 1 and class 2 and *T* is the threshold value.

The above function without further assumptions represents the quadratic discriminate function. If the covariance matrices $\Sigma_{1}=\Sigma_{2}=\Sigma$, then the discriminant function simplifies to a dot product.

(18) x.y>constant,

where $x=\Sigma^{-1}\left(\mu_{1}-\mu_{2}\right)$, $\mathrm{constant}=\frac{1}{2}\left(T-\mu_{1}^{T}\Sigma \mu_{1}-\mu_{2}\Sigma \mu_{2}^{T}\right)$. This decision rule represents the classification based on linear discriminant.

The parameters used for linear discriminant analysis (LDA) and quadrature discriminant analysis (QDA) classifiers are given below in Table 3.

Ridge parameters in the discriminant analysis classifiers reduce the overfitting problem by penalizing the large quantity coefficients. In our work, we use the default values as given in Table 3.

**J48 Classifier:** Recently, decision tree-based algorithms have become popular in machine learning strategies. In practice, J48 is an execution of popular C 4.5 algorithms proposed by Quinlan [53]. According to this algorithm, the decision process involves the construction of a tree based on the feature splitting. The superiority of matching y to a class label $\omega_{k}\in \omega$ depends on the choice of feature splitting based on the value of information gain.

Information gain is measured with the help of difference entropy as the difference between the entropy of the central node to the sum of entropies of the leaf nodes. It measures how well a given feature splits the training data under its class label. A feature node having high information gain is preferred.

**J48 Consolidated (J48-C) Classifier:** It is a consolidated version of C 4.5 classifier. “J48 consolidated’’ is an implementation of a consolidated tree’s construction algorithm, proposed by Arbelaiz et al. [54] in WEKA. The basic idea is building a single tree using several subsamples. In each iteration, we will find a better feature using information gain content similar to J48. After finding the best feature split, all the subsamples are divided using the same features. More details can be found in [54].The parameters used for J48 and J48-C classifiers are given in below Table 4.

J48 and J48-C classifiers are decision tree classifiers in which tree splitting criteria play a significant role. The above-mentioned parameters determine the growth and direction of the tree structures that influence the final model accuracy. Sub-tree raising considers raising of a sub-tree when pruning is enabled. The minimum number of objects determines the number of instances per leaf. Minimum description length (MDL) correction is a statistical measure like information gain to identify the best split tree. The number of folds determines the data used for error reduce pruning; here, one fold is for pruning and the other folds for building the tree.

All the parameters are fixed based on the final results. All the details of the parameters can be found in WEKA 3.9 version [56].

## 3. Results

In this work, we are classifying the five classes: N, V, S, F, Q. The training set is constructed with the array of records as DS1 = [101, 106, 108, 109, 112, 114, 115, 116, 118, 119, 122, 124,201, 203, 205, 207, 208, 209, 215, 220, 223, 230], and DS2 = [100, 103, 105, 111, 113, 117, 121, 123, 200, 202, 210, 212, 213, 214, 219, 221, 222, 228, 231, 232, 233, 234]. Here, the numerical values represent the patient record number. Four records (102, 104, 107 and 217) having paced beats are exempted from both DS1 and DS2 data sets.

We start with scatter plots for justifying the choice of features in discriminating against the heartbeats. Individual performance of five classifiers naïve Bayes, LDA, QDA, J48, and J48 consolidated is presented, and analysis using a voting scheme with various combinations of these classifiers is considered. The performance is illustrated for each combination. We used the WEKA 3.9 version (University of Waikato, New Zealand) [56] for implementing the classification algorithms and scatter plots. Data pre-processing and feature extraction is implemented using MATLAB 2018a (Mathworks, MA, USA). All the experiments are carried out in Windows 8, 8 GB RAM, and 64-bit operating system.

### The Performance Measures

An algorithm’s efficiency can be validated with appropriate performance measures. In this work, Sensitivity (SEN), False Positive Rate (FPR), Positive Predictive Value (PPV), and Overall Accuracy (OA) are used as performance measures to compare with the state-of-the-art methods, following the AAMI recommendations. The confusion matrix required for calculating these measures is given in Table 5. For V and S classes, the measures are calculated as per [31]. For remaining classes, we followed [57].

Performance measure from Table 5 can be calculated as follows:

The sum measures of row-wise and column-wise calculations are:

(19) $$\begin{array}{ccc} R_{N}=N_{n}+N_{v}+N_{s}+N_{f}+N_{q}; & \; & C_{N}=N_{n}+V_{n}+S_{n}+F_{n}+Q_{n};\\ R_{V}=V_{n}+V_{v}+V_{s}+V_{f}+V_{q}; & \; & C_{V}=N_{v}+V_{v}+S_{v}+F_{v}+Q_{v};\\ R_{S}=S_{n}+S_{v}+S_{s}+S_{f}+S_{q}; & \; & C_{S}=N_{s}+V_{s}+S_{s}+F_{s}+Q_{s};\\ R_{F}=F_{n}+F_{v}+F_{s}+F_{f}+F_{q}; & \; & C_{F}=N_{f}+V_{f}+S_{f}+F_{f}+Q_{f};\\ R_{Q}=Q_{n}+Q_{v}+Q_{s}+Q_{f}+Q_{q}; & \; & C_{Q}=N_{q}+V_{q}+S_{q}+F_{q}+Q_{q};\\ R=R_{N}+R_{V}+R_{S}+R_{F}+R_{Q}; & \; & C=C_{N}+C_{V}+C_{S}+C_{F}+C_{Q};\\ R=C. & \; & \; \end{array}$$

The false-positive and false negative values for each class are defined as below:

(20) $$\begin{array}{ccc} FN_{N}=R_{N}-N_{n}; & \; & FP_{N}=C_{N}-N_{n};\\ FN_{V}=R_{V}-V_{v}; & \; & FP_{V}=C_{V}-\left(V_{v}+F_{v}+Q_{v}\right);\\ FN_{S}=R_{S}-S_{s}; & \; & FP_{S}=C_{S}-\left(S_{s}+Q_{s}\right);\\ FN_{F}=R_{F}-F_{f}; & \; & FP_{F}=C_{N}-F_{f};\\ FN_{Q}=R_{Q}-Q_{q}; & \; & FP_{Q}=C_{N}-Q_{q}. \end{array}$$

The other useful measures, true positives, and negatives can be calculated for Classes N,V,S,F,Q:

(21) $$\begin{array}{ccc} TP_{N}=N_{n}; & \; & TN_{N}=R-\left(R_{N}+C_{N}-N_{n}\right);\\ TP_{N}=V_{v}; & \; & TN_{V}=R-\left(R_{V}+C_{V}-V_{v}\right);\\ TP_{S}=S_{s}; & \; & TN_{S}=R-\left(R_{S}+C_{S}-S_{s}\right);\\ TP_{F}=F_{f}; & \; & TN_{F}=R-\left(R_{F}+C_{F}-F_{f}\right);\\ TP_{Q}=Q_{q}; & \; & TN_{Q}=R-\left(R_{Q}+C_{Q}-Q_{q}\right). \end{array}$$

The performance measures are given by

$$\mathrm{SEN}=\frac{TP}{TP+FN};\mathrm{FPR}=\frac{FP}{TN+FP};\mathrm{PPV}=\frac{TP}{TP+FP};\mathrm{OA}=\frac{TP_{N}+TP_{V}+TP_{S}+TP_{F}+TP_{Q}}{R},$$

where TP=True Positive,TN=True Negative,FP=False Positive,andFN=False Negative.

**Figure 2:**
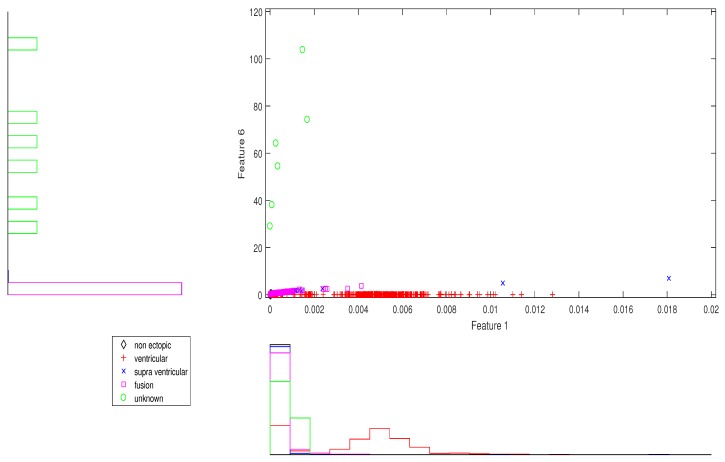
Scatter plot with marginal histogram for CUM2 (IMF1) vs. norm entropy (IMF1).

**Figure 3:**
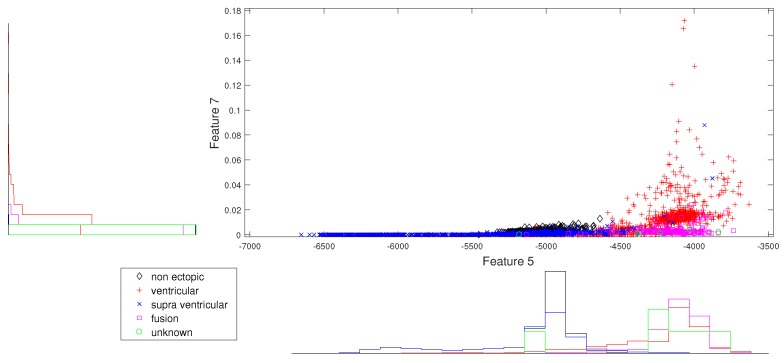
Scatter plot with marginal histogram for log entropy (IMF1) vs. CUM2 (IMF1).

**Figure 4:**
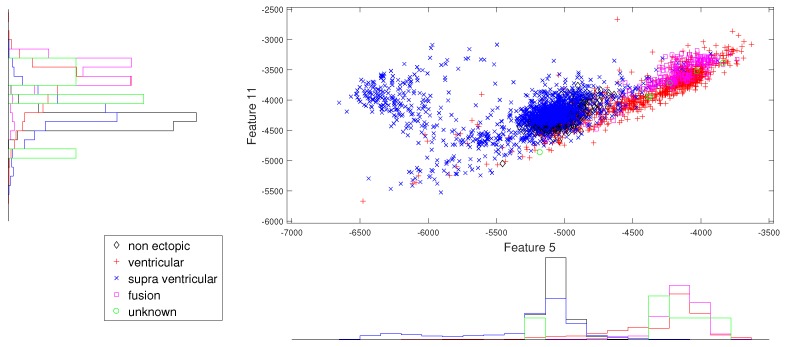
Scatter plot with marginal histogram for log entropy (IMF1) vs. log entropy (IMF2).

**Figure 5:**
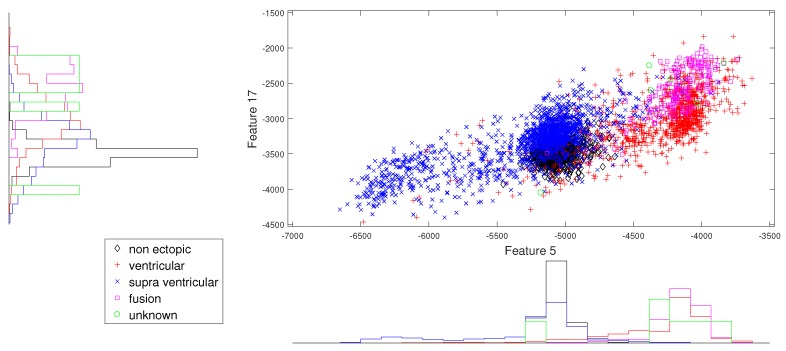
Scatter plot with marginal histogram for log entropy (IMF1) vs. log entropy (IMF3).

**Figure 6:**
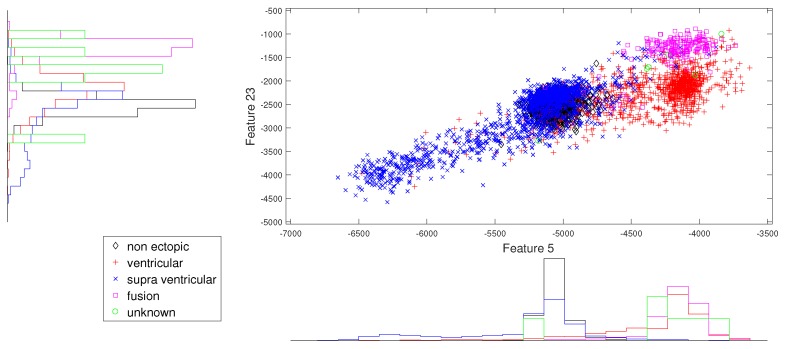
Scatter plot with marginal histogram for log entropy (IMF1) vs. log entropy (IMF4).

We present the scatter plots with marginal histograms on the testing data set DS2, for features in the two-dimensional feature space. These scatter plots reveal how different features spread in feature space, thereby revealing the relationship between different heartbeat classes. Figure 2 shows the two-dimensional scatter plot between cumulant 2 of IMF1 to norm entropy value of IMF2. In this plot, we can observe that N and V beats are dominantly spread across space. In addition, the histogram plots also reveal the good discrimination between N, V, and Q classes out of the five classes. The next plot from Figure 3 gives the relation between the log energy entropy of IMF1 to cumulant 2 of IMF1. In this figure, we can see the spreading of N, V, S, and F classes in the space. In particular, this space provides good discrimination between N, V, and S classes. From Figure 4, Figure 5 and Figure 6, we can observe that log energy entropy values extracted from different IMFs provide a good perception of N, V, and S classes.

**Figure 7:**
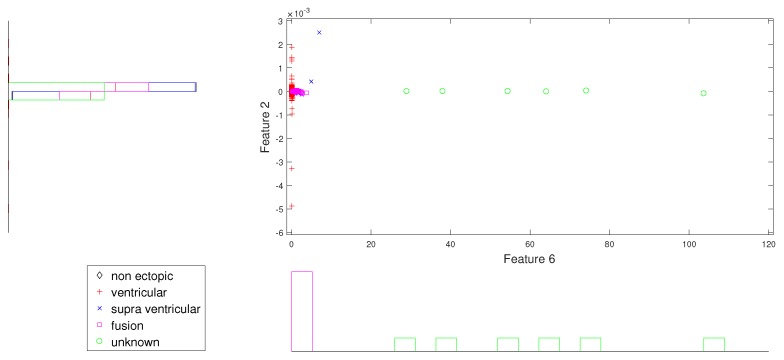
Scatter plot with marginal histogram for norm entropy (IMF1) vs. CUM2 (IMF1).

**Figure 8:**
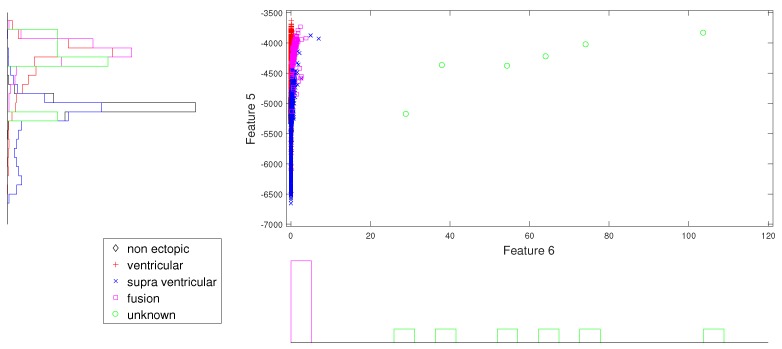
Scatter plot with marginal histogram for norm entropy (IMF1) vs. log entropy (IMF1).

In the same way, Figure 7 and Figure 8 give better discrimination of Q beats, which are very rare indeed. In these figures, the characteristic feature is the norm entropy. In addition, different combinations of features with norm entropy reveal different class spreads and discrimination capabilities. As a whole, we can say that the combinations of selected features from different IMFs can predict the required hypothesis.

After dividing the training and testing feature sets, we need to learn a model for classification. In this work, we used an ensemble learner for classification. Ensemble classifiers use multiple learning algorithms and combine all the decisions. It can be more accurate than the individual classifiers. The main advantage of the ensemble classifiers is that we can achieve low bias error and low variance error. Ensembles using multiple trained (high variance/ low bias) models can average out of the variance, leaving just the bias. In addition, ensemble classifiers are preferred for imbalanced datasets. Our DS1 and DS2 datasets are highly imbalanced with majority N group class and minor F and Q classes. Therefore, in this work, we used a voting scheme based on product rule to ensemble the classifiers. The individual classifier performance on DS2 (testing data) is presented in Table 6 and Table 7. Confusion matrices calculated for LDA, QDA, naïve Bayes, J48 and J48-C classifiers are shown in Table 6. The performance measures for the corresponding matrices based on Table 5 are presented in Table 7.

From this table, we can see that each classifier yields different prediction. LDA and J48 give better classification for the N and V classes. It is an understandable phenomenon because of the dominating number of examples in N and V. LDA and J48-C provides better discrimination to Q group. The other classifiers J48-C and naïve Bayes are providing better SEN results for F group. Finally, S class is predicted accurately by J48-C and QDA classifiers. The other important point is, although all classifiers yield better results for the specific group of categories, the OA is dominated by the N class discrimination. Therefore, it is noticeable that OA is no longer a useful performance measure for imbalanced data classification.

In Table 8, the confusion matrix after combining J48, LDA, and naïve Bayes classifiers using the voting scheme is presented. The corresponding performance measures are demonstrated in Table 9. From the results, it is evident that this combination yields better results for N, V, F, and Q classes and average result for S class. The critical point is N, and S classes have more morphological similarities. Therefore, individual classifiers are giving complementary results for N and S. However, this ensemble selection enhances the prediction generalization for both classes.

Similarly, we performed ensemble voting for different combinations and the results are presented in Table 10, Table 11, Table 12, Table 13, Table 14 and Table 15. Each combination provides various enhanced results in some aspects.

As mentioned earlier, the dataset is dominated by N, V, and S classes, respectively. The F and Q classes are sporadic. Therefore, in some works, only N, V, and S classes are considered for classification. We provide the results for such schemes in Table 16, Table 17, Table 18, Table 19, Table 20, Table 21, Table 22, Table 23, Table 24 and Table 25. Here, first results are also presented for individual classifiers; later, the ensemble voting scheme is performed on different combinations of classifiers. Each one gives better classifications than individual classifiers.

## 4. Discussion

This section contains a discussion on simulating the proposed methodology illustrated in Figure 1. In this work, we employ an adaptive non-stationary and nonlinear decomposition method, namely ICEEMD, to analyze the ECG heartbeats. ICEEMD produces a local and entirely data-driven separation of a signal in the form of fast and slow oscillations called IMFs. The main advantage of ICEEMD is that it successfully avoids the spurious nodes and reduces the amount of noise in the modes.

Later, six nonlinear morphological features: higher-order cumulants, log, Shannon energy, and norm entropies are extracted from the first six IMFs of each heartbeat, to generate a 36×1 feature vector. Then, these feature vectors are divided based on training and testing sets DS1 and DS2 as specified above. Statistics (median and interquartile range) of these features for each class are presented in Table 26. Variation of attributes corresponding to different heartbeats can be observed from this table.

In Table 6, we presented the individual performance of various classifiers on the given problem. Here, all classifier models offer separate results for all the classes. Each model performs well for a specific class or classes. However, it fails in providing the overall better performance. For example, the S class contains 1837 beats, the J48, LDA, and naïve Bayes are predicting 51, 2, 1516 beats, respectively. Whenever we combine these three models using the voting scheme as shown in Table 12, this combined model identified 779 beats correctly. The voting scheme uses the product of probabilities rule. In this scheme, it is assumed that each model representation for a given class is statistically independent. It is because of the different representation capabilities of each model. From this, a final decision rule is formed as described in Section 2.4. This decision rule quantifies the probability of class choice from combined hypothesis models and the same type of results we can observe for other classes. In this work, we implemented four voting schemes with different classifier combinations. Each combination again provides different but better results than individual classifier models. The proposed combinations of classifier details are given in Table 27.

### 4.1. Comparative Analysis

To assess the performance of our proposed methodology, we compared our results with the existing methods in the literature. Comparisons are presented in Table 28 and Table 29. The features and classification schemes employed by various researchers listed in Table 28 and Table 29 are given in Table 27. In Table 28, we compare our four sets of voting schemes with the works which followed AAMI recommendations based on [31] division scheme. In addition, Table 29 shows the performance measure comparison with the literature on only N, V, and S classification. In Table 28, our proposed methods yield almost similar performance compared to the state-of-the-art for N, S, and V classes; however, in case of F and Q classes, our proposed work one and four outperforms the other compared methods. From Table 29, it is evident that our proposed methods one and three are efficiently distinguished the classes N, S, and V. The best results of our method are highlighted in bold. Overall, the measures of our work are appreciable compared with other approaches.

### 4.2. Limitation and Future Scope

Despite the proposed method giving significant results, the performance of the S class is still limited when compared to other classes. Similar behavior is observed in other state-of-the-art methods. Hence, there is a need to explore a new set of attributes and learning algorithms to improve this. In addition, incorporating other physiological signals such as blood pressure, plethysmographic signals along with ECG may improve the description of “heart functioning.”

## 5. Conclusions

In this work, we implemented a computer-aided inter-patient heartbeat classification algorithm. We employed a nonlinear decomposition method called ICEEMED, to extract some important information lying in ECG. Later, HOS and entropy measures are calculated on the modes obtained after ICEEMD and used as features. Class imbalance is one of the critical challenges in medical diagnosis. We addressed this issue by utilizing the voting scheme as the learning model. The extracted features are then fed to this model for classification. To design this model, naïve Bayes, linear and quadratic discriminating functions, J48 and J48 consolidated classifiers are explored. The proposed method showed promising results compared to state-of-the-art techniques. Our method opens new frontiers to the successful identification of rare heartbeat groups enabling a real-time heart monitoring system.

## Figures and Tables

**Table 1:** Details of the number of beats selected from each group of AAMI classes.

AAMI Classes	MIT-BIH Heartbeats	Total Data	Training (DS1)	Testing (DS2)
N	Normal, left and right bundle branch block	83,761	41,746	42,015
	atrial and nodal escape beats				
S	atrial premature contraction, Aberrated atrial,	2614	777	1837
	supra ventricular and junctional premature beats				
V	premature ventricular contraction, ventricular flutter	6893	3787	3106
	and escape beats				
F	fusion of ventricular and normal	526	266	260
	beats				
Q	paced, unclassifiable,	12	6	6
	fusion of paced and normal beats				

**Table 2:** Naïve Bayes Classifier parameters used in this work.

Parameters	Naïve Bayes
Use Kernel Estimator	False
Use supervise Discretization	True

**Table 3:** LDA and QDA classifier parameters used in this work.

Parameters	LDA	QDA
Ridge	$1.0\times 10^{-6}$	$1.0\times 10^{-6}$

**Table 4:** J48 and J48-C classifier parameters used in this work.

Parameters	J48	J48-C
Minimum Objects	1000	1000
Use MDL correction	True	True
Number of folds	3	3
Sub-tree raising	True	True

Details of the parameters can be find in WEKA 3.9 version [56].

**Table 5:** Confusion matrix.

			Predicted Labels			
Actua Labels	N	V	S	F	Q	Sum
N	$N_{n}$	$N_{v}$	$N_{s}$	$N_{f}$	$N_{q}$	$R_{N}$
V	$V_{n}$	$V_{v}$	$V_{s}$	$V_{f}$	$V_{q}$	$R_{V}$
S	$S_{n}$	$S_{v}$	$S_{s}$	$S_{f}$	$S_{q}$	$R_{S}$
F	$F_{n}$	$F_{v}$	$F_{s}$	$F_{f}$	$F_{q}$	$R_{F}$
Q	$Q_{n}$	$Q_{v}$	$Q_{s}$	$Q_{f}$	$Q_{q}$	$R_{Q}$
Sum	$C_{N}$	$C_{V}$	$C_{S}$	$C_{F}$	$C_{Q}$	R/C

**Table 6:** Confusion matrices for LDA, QDA, naïve Bayes, J48, and J48-C classifiers.

LDA						QDA					
	N	V	S	F	Q		N	V	S	F	Q
N	40,842	33	47	1093	0		2777	266	36,394	2578	0
V	319	2787	0	0	0		5	3101	0	0	0
S	1831	1	2	3	0		95	56	1675	11	0
F	171	0	0	89	0		91	64	20	85	0
Q	0	0	0	0	6		0	6	0	0	0
**naïve Bayes**						**J48**					
	N	V	S	F	Q		N	V	S	F	Q
N	36,222	745	997	3289	762		41,801	214	0	0	0
V	930	1874	52	231	19		363	2743	0	0	0
S	1321	3	133	27	353		1819	18	0	0	0
F	8	0	2	245	5		259	1	0	0	0
Q	1	0	0	4	1		5	1	0	0	0
**J48-C**											
				N	V	S	F	Q			
			N	0	6941	28,966	5290	818			
			V	0	2205	894	6	1			
			S	0	24	1785	19	9			
			F	0	30	23	191	16			
			Q	0	0	0	0	6			

**Table 7:** Performance measures for LDA, QDA, naïve Bayes, J48 and J48-C classifiers.

LDA			QDA				
OA = 92.60	SEN%	FPR%	PPV%	OA = 16.20	SEN%	FPR%	PPV%
N	97.2	44.6	94.6		6.6	3.7	93.6
V	89.7	0.1	98.8		99.8	0.9	88.8
S	0.1	0.1	4.1		91.2	80.2	4.4
F	34.2	2.3	7.5		32.7	5.5	3.2
Q	100	0	100		0	0	0
**naïve Bayes**			**J48**				
OA = 81.47	SEN%	FPR%	PPV%	OA = 94.32	SEN%	FPR%	PPV%
N	86.2	43.4	94.1		99.5	47	94.5
V	60.3	1.7	71.5		88.3	0.5	92.1
S	7.2	2.3	11.2		0	0	0
F	94.2	7.6	6.5		0	0	0
Q	16.7	2.4	0.1		0	0	0
**J48-C**							
			OA = 8.86	SEN%	FPR%	PPV%	
			N	0	0	0	
			V	71.0	15.9	24.0	
			S	97.2	65.8	5.6	
			F	73.5	11.3	3.5	
			Q	100	1.8	0.7	

**Table 8:** Confusion matrix for combining J48, LDA, and naïve Bayes classifiers using a Voting scheme.

Voting (J48, LDA, naïve Bayes)					
	N	V	S	F	Q
N	39,542	53	489	1931	0
V	395	2708	3	0	0
S	1473	1	353	10	0
F	22	5	1	232	0
Q	0	0	0	0	6

**Table 9:** Performance measures for combining J48, LDA, and naïve Bayes classifiers using a Voting scheme.

Voting (J48, LDA, naïve Bayes)			
OA = 90.71	SEN%	FPR%	PPV%
N	94.1	36.3	95.4
V	87.2	0.1	97.9
S	19.2	1.1	41.7
F	89.2	4.1	10.7
Q	100	0	100

**Table 10:** Confusion matrix for combining J48, naïve Bayes, and QDA classifiers using a voting scheme.

Voting ( J48, naïve Bayes, QDA)					
	N	V	S	F	Q
N	35,629	253	3836	2297	0
V	11	3095	0	0	0
S	1188	11	624	14	0
F	28	61	11	160	0
Q	0	6	0	0	0

**Table 11:** Performance measures for combining J48, naïve Bayes, and QDA classifiers using a Voting scheme.

Voting ( J48, naïve Bayes, QDA)			
OA = 83.6	SEN%	FPR%	PPV%
N	84.8	23.6	96.7
V	99.6	0.8	90.3
S	34	8.5	14
F	61.5	4.9	6.5
Q	0	0	0

**Table 12:** Confusion matrix for combining J48-C, naïve Bayes, and QDA classifiers using a Voting scheme.

Voting (J48-C, naïve Bayes, QDA)					
	N	V	S	F	Q
N	29,730	255	9089	2491	0
V	8	3098	0	0	0
S	1031	8	779	19	0
F	21	61	12	166	0
Q	0	6	0	0	0

**Table 13:** Performance measures for combining J48-C, naïve Bayes, and QDA classifiers using a Voting scheme.

Voting (J48-C,naïve Bayes, QDA)			
OA = 71.51	SEN%	FPR%	PPV%
N	70.8	20.3	96.6
V	99.7	0.7	90.4
S	42.4	20.1	7.9
F	63.8	6.3	5.3
Q	0	0	0

**Table 14:** Confusion matrix for combining J48-C, naïve Bayes, and LDA classifiers using a Voting scheme.

Voting (J48-C, naïve Bayes, LDA)					
	N	V	S	F	Q
N	38,329	66	942	2678	0
V	553	2532	20	1	0
S	1405	2	416	14	0
F	20	4	1	235	0
Q	0	0	0	0	6

**Table 15:** Performance measures for combining J48-C, naïve Bayes, and LDA classifiers using a Voting scheme.

Voting (J48-C,naïve Bayes, LDA)			
OA = 87.91	SEN%	FPR%	PPV%
N	91.2	38	95.1
V	81.5	0.2	97.2
S	22.6	2.1	30.2
F	90.4	5.7	8
Q	100	0	100

**Table 16:** Confusion matrices for various classifiers (N, V, and S classes).

LDA			QDA				
	N	V	S		N	V	S
N	41,875	27	113		4569	480	36,966
V	280	2826	0		5	3101	0
S	1835	0	2		105	56	1676
**naïve Bayes**			**J48**				
	N	V	S		N	V	S
N	37,385	2968	1662		41,998	17	0
V	922	2120	64		1095	2011	0
S	1336	18	483		1837	0	0
**J48-C**							
				N	V	S	
			N	34,999	7016	0	
			V	651	2455	0	
			S	1585	252	0	

**Table 17:** Performance measures for various classifiers (N,V,S classes).

LDA			QDA				
OA = 95.19	SEN%	FPR%	PPV%	OA = 19.90	SEN%	FPR%	PPV%
N	99.7	42.8	95.2		10.9	2.2	97.6
V	91.0	0.1	99.1		99.8	1.2	85.3
S	0.1	0.3	1.7		91.2	81.9	4.3
**naïve Bayes**			**J48**				
OA = 85.15	SEN%	FPR%	PPV%	OA = 93.71	SEN%	FPR%	PPV%
N	89	45.7	94.3		100	59.3	93.5
V	68.3	6.8	41.5		64.7	0	99.2
S	26.3	3.8	21.9		0	0	0
**J48-C**							
			OA = 79.76	SEN%	FPR%	PPV%	
			N	83.3	45.2	94	
			V	79	16.6	25.2	
			S	0	0	0	

**Table 18:** Confusion matrix for combining J48, LDA, and naïve Bayes classifiers using a Voting scheme (N,S,V).

Voting ( J48, LDA, naïve Bayes)			
	N	V	S
N	40,918	361	736
V	205	2897	4
S	1469	3	365

**Table 19:** Performance measures for combining J48, LDA, and naïve Bayes classifiers using Voting scheme (N,S,V).

Voting ( J48, LDA, naïve Bayes)			
OA = 94.08	SEN%	FPR%	PPV%
N	97.4	33.9	96.1
V	93.3	0.8	88.8
S	19.9	1.6	33

**Table 20:** Confusion matrix for combining J48, naïve Bayes, and QDA classifiers using Voting scheme (N,S,V).

Voting ( J48, naïve Bayes, QDA)			
	N	V	S
N	37,421	574	4020
V	12	3094	0
S	1203	8	626

**Table 21:** Performance measures for combining J48, naïve Bayes, and QDA classifiers using Voting scheme (N,S,V).

Voting ( J48, naïve Bayes, QDA)			
OA = 87.61	SEN%	FPR%	PPV%
N	89.1	24.6	96.9
V	99.6	1.3	84.2
S	34.1	8.9	13.5

**Table 22:** Confusion matrix for combining J48-C, naïve Bayes, and QDA classifiers using Voting scheme (N,S,V).

Voting ( J48-C, naïve Bayes, QDA)			
	N	V	S
N	32011	598	9406
V	7	3099	0
S	1062	8	767

**Table 23:** Performance measures for combining J48-C, naïve Bayes, and QDA classifiers using Voting scheme (N,S,V).

Voting ( J48-C, naïve Bayes, QDA)			
OA = 76.40	SEN%	FPR%	PPV%
N	76.2	21.6	96.8
V	99.8	1.4	83.6
S	41.8	20.8	7.5

**Table 24:** Confusion matrix for combining J48-C, naïve Bayes, and LDA classifiers using Voting scheme (N,S,V).

Voting ( J48-C, naïve Bayes, LDA)			
	N	V	S
N	40,248	688	1079
V	359	2735	12
S	1407	2	428

**Table 25:** Performance measures for combining J48-C, naïve Bayes, LDA classifiers using Voting scheme (N,S,V).

Voting ( J48-C, naïve Bayes, LDA)			
OA = 92.44	SEN%	FPR%	PPV%
N	95.8	35.7	95.8
V	88.1	1.6	79.9
S	23.3	2.4	28.2

**Table 26:** Median ± interquartile range of features extracted on DS1.

Feature Number	Features	N	S	V	F	Q	
1	CUM2(IMF1)	1.0 $\;\times \;\;10^{-6}$± 4.0 $\;\times \;\;10^{-6}$	8.0 $\;\times \;\;10^{-6}$± 4.03 $\;\times \;\;10^{-5}$	7.50 $\;\times \;\;10^{-5}$± 0.00045775	0.000162 ± 0.000326	0.0001405 ± 0.009121	
2	CUM3(IMF1)	0 ± 0	0 ± 0	0 ± 2.0 $\;\times \;\;10^{-6}$	0 ± 5.0 $\;\times \;\;10^{-6}$	0 ± 0.000313	
3	CUM4(IMF1)	0 ± 0	0 ± 0	1.0 $\;\times \;\;10^{-6}$± 2.58 $\;\times \;\;10^{-5}$	2.50 $\;\times \;\;10^{-6}$± 1.1$\;\times \;10^{-5}$	4.5$\;\times \;10^{-6}$± 0.006113	
4	Shan(IMF1)	0.004769 ± 0.009686	0.017643 ± 0.06530625	0.112159 ± 0.4199265	0.216149 ± 0.330006	0.153638 ± 1.47632	
5	log(IMF1)	−4792.4962 ± 379.828	−4735.1172 ± 286.978	−4532.44 ± 467.507	−4358.371 ± 271.824	−4604.445 ± 1783.20	
6	norm(IMF1)	0.1004 ± 0.0726	0.1538 ± 0.1719	0.0815 ± 0.05794	0.5541 ± 0.5434	18.1790 ± 20.55612	
7	CUM2(IMF2)	0.001 ± 0.00347	0.00109 ± 0.0035	0.00028 ± 0.00137	0.00107 ± 0.00143	0.00411 ± 0.0134	
8	CUM3(IMF2)	8.0$\;\times \;10^{-6}$± 5.20$\;\times \;10^{-5}$	4.0$\;\times \;10^{-6}$± 5.10$\;\times \;10^{-5}$	0 ± 6.0$\;\times \;10^{-6}$	5.0$\;\times \;10^{-6}$± 2.10$\;\times \;10^{-5}$	0 ± 0.000387	
9	CUM4(IMF2)	1.50$\;\times \;10^{-5}$± 0.000185	2.10$\;\times \;10^{-5}$± 0.000128	2.0$\;\times \;10^{-6}$± 4.20$\;\times \;10^{-5}$	1.60$\;\times \;10^{-5}$± 5.20$\;\times \;10^{-5}$	0.00046 ± 0.002581	
10	Shan(IMF2)	1.3074 ± 2.9180	1.3675 ± 3.4250	0.431 ± 1.429	1.4044 ± 1.44285	2.90440 ± 6.76104	
11	log(IMF2)	−4055.72 ± 403.632	−4045.57 ± 565.833	−4064.90 ± 479.11	−3788.21 ± 266.57	−4023.39 ± 1366.58	
12	norm(IMF2)	2.41 ± 3.81	2.57 ± 4.95	2.744 ± 1.768	2.69 ± 2.04	18.17 ± 20.556	
13	CUM2(IMF3)	0.0069 ± 0.0097	0.00534 ± 0.009013	0.00443 ± 0.00936	0.011997 ± 0.00909	0.004148 ± 0.008015	
14	CUM3(IMF3)	−7.80$\;\times \;10^{-5}$± 0.000322	−8.60$\;\times \;10^{-5}$± 0.0003605	−3.10$\;\times \;10^{-5}$± 0.000246	−0.00019 ± 0.00062	8.95$\;\times \;10^{-5}$± 0.000266	
15	CUM4(IMF3)	0.000235 ± 0.000863	0.000104 ± 0.0006515	0.000117 ± 0.000747	0.0005255 ± 0.000767	0.0001945 ± 0.000396	
16	Shan(IMF3)	6.3075515 ± 6.075866	5.532014 ± 6.254886	4.570122 ± 5.93904725	10.002892 ± 5.49255	3.8205085 ± 6.888019	
17	log(IMF3)	−3114.4106 ± 443.70730	−3071.71175 ± 705.504	−3178.659 ± 531.3571	−2731.2660 ± 467.4692	−3341.5431 ± 891.0573	
18	norm(IMF3)	9.3564 ± 7.6444	8.69133 ± 8.37546	7.585 ± 4.1465	14.85 ± 7.440	18.179 ± 20.55	
19	CUM2(IMF4)	0.013817 ± 0.0210	0.01233 ± 0.01653	0.0215 ± 0.03611	0.0370 ± 0.0297	0.0069 ± 0.0102	
20	CUM3(IMF4)	−0.00012 ± 0.00081	−6.80$\;\times \;10^{-5}$± 0.000469	−0.00014 ± 0.001305	−0.001542 ± 0.00207	0 ± 0.00039	
21	CUM4(IMF4)	0.000147 ± 0.000734	4.60$\;\times \;10^{-5}$± 0.0003225	0.000404 ± 0.00189	0.00027 ± 0.00124	0.000138 ± 0.00018	
22	Shan(IMF4)	13.05585 ± 12.76457	12.2365 ± 11.295	16.104 ± 15.135	25.934 ± 15.110	7.113 ± 10.471	
23	log(IMF4)	−2150.494 ± 533.542	−2143.567 ± 611.308	−2206.943 ± 794.75	−1614.17 ± 480.99	−2593.22 ± 1271.81	
24	norm(IMF4)	19.367 ± 15.509	18.686 ± 14.240	16.26 ± 9.166	35.59 ± 20.941	18.179 ± 20.556	
25	CUM2(IMF5)	0.0084 ± 0.018	0.0066 ± 0.0156	0.033 ± 0.0648	0.056 ± 0.058	0.0075 ± 0.0149	
26	CUM3(IMF5)	1.0$\;\times \;10^{-6}$± 0.00017	0 ± 0.000103	−1.0$\;\times \;10^{-6}$± 0.00146	1.40$\;\times \;10^{-5}$± 0.0022	0 ± 0.000405	
27	CUM4(IMF5)	−7.00$\;\times \;10^{-6}$± 0.00012	−6.0$\;\times \;10^{-6}$± 0.000127	−3.0$\;\times \;10^{-5}$± 0.00193	−0.00236 ± 0.00621	−5.50$\;\times \;10^{-6}$± 0.000287	
28	Shan(IMF5)	10.358 ± 15.6374	8.68112 ± 14.823	25.882 ± 29.195	39.2240 ± 25.737	8.992 ± 16.965	
29	log(IMF5)	−1917.009 ± 593.83	−1992.177 ± 872.920	−1567.422 ± 918.993	−1216.140± 489.3834	−2179.500 ± 1495.143	
30	norm(IMF5)	17.118 ± 19.0735	14.983 ± 19.2726	19.495 ± 14.659	52.96 ± 32.190	18.179 ± 20.556	
31	CUM2(IMF6)	0.0040 ± 0.0092	0.0028 ± 0.00723	0.0184 ± 0.04606	0.0571 ± 0.0827	0.0073 ± 0.01072	
32	CUM3(IMF6)	1.0$\;\times \;10^{-6}$± 0.00010	0 ± 6.80$\;\times \;10^{-5}$	2.0$\;\times \;10^{-6}$± 0.0011117	8.0$\;\times \;10^{-6}$± 0.00527	0 ± 0.0002	
33	CUM4(IMF6)	−1.50$\;\times \;10^{-5}$± 0.00011	-8.0$\;\times \;10^{-5}$ 6± 6.25$\;\times \;10^{-5}$	−0.00024 ± 0.00205	−0.00389± 0.01428	−4.40$\;\times \;10^{-5}$± 0.00017	
34	Shan(IMF6)	6.50405 ± 11.11697	5.26119 ± 9.9055	20.0112 ± 31.26347	44.49 ± 39.5131	10.284 ± 13.481	
35	log(IMF6)	−1903.169 ± 637.510	−1983.32 ± 713.457	−1470.196 ± 744.176	−1062.5 ± 546.13	−1682.363 ± 1296.591	
36	norm(IMF6)	13.0914 ± 15.1495	11.4410 ± 14.268	10.566 ± 12.262	57.357 ± 46.317	18.179 ± 20.556	

Note: CUM2-second order cumulnat, CUM3-third order cumulant, CUM4-Fourth order cumulant, Shan-Shannon entropy, log-Log entropy, norm- norm entropy.

**Table 27:** Methodology description of recent state-of-the-art compared in our work.

Literature	Feature Extraction	Classification
[58]		
method-1	R–R intervals	Optimum Path Forest (OPF)
method-2	Wavelet based features	OPF
method-3	Mean, standard deviation and average power of wavelet sub-band	OPF
method-4	Auto correlation and energy ratio of wavelet bands	OPF
method-5	Fast-ICA	OPF
method-6	(Wavelet+ICA+RR interval)	OPF
[59]	(ECG+VCG) complex network based features	SVM
[42]	Wavelet packet decomposition based entropy features	Random Forest
[60]		
method-1	Wavelet based features	Hierarchical Classification (tree approach)
method-2	Mean, standard deviation and average power of wavelet sub-band	Hierarchical Classification (tree approach)
method-3	Auto correlation and energy ratio of wavelet bands	Hierarchical Classification (tree approach)
method-4	Fast-ICA	Hierarchical Classification (tree approach)
method-5	(Wavelet+ICA+RR interval)	Hierarchical Classification (tree approach)
[61]	Temporal Vectrcardiogram(TCG) based features	SVM
[62]	A combination of projected features	
	(features derived from the projected matrix and DCT) and RR intervals	SVM
[63]	TCG feature selection by PSO	SVM
**proposed work**		
method-1	Entropy and statistical features calculated on ICEEMD modes	Voting ( J48, LDA, naïve Bayes)
method-2	Entropy and statistical features calculated on ICEEMD modes	Voting ( J48, QDA, naïve Bayes)
method-3	Entropy and statistical features calculated on ICEEMD modes	Voting ( J48-C, QDA, naïve Bayes)
method-4	Entropy and statistical features calculated on ICEEMD modes	Voting ( J48-C, LDA, naïve Bayes)

**Table 28:** Performance comparison with recent literature (N, S, V, F, and Q classes).

Literature	N	S	V	F	Q
	SEN/FPR/PPV	SEN/FPR/PPV	SEN/FPR/PPV	SEN/FPR/PPV	SEN/FPR/PPV
[58]					
method-1	84.5/-/-	1.0/-/-	77.7/-/-	38.4/-/-	0/-/-
method-2	86.4/-/-	2.3/-/-	40.8/-/-	0.5/-/-	0/-/-
method-3	84.8/-/-	18.3/-/-	77.8/-/-	7.5/-/-	0/-/-
method-4	92.5/-/-	3.0/-/-	61.8/-/-	16.8/-/-	0/-/-
method-5	95.7/-/-	17.7/-/-	74.7/-/-	3.9/-/-	0/-/-
method-6	93.2/-/-	12.1/-/-	85.5/-/-	18.3/-/-	0/-/-
[59]	89.3/25.2/96.6	38.6/6.7/18	81.2/4.9/53.6	0/0/0	0/0/0
[42]	94.67/3.92/99.73	20/3.69/0.16	94.20/0.71/89.78	50/0.78/0.52	0/0/0
[60]					
method-1	92.3/22.2/97.1	28.5/2.6/29.6	83.5/5.51/51.2	19.1/1.07/12.3	0/0/-
method-2	93.6/57.1/93.0	0.49/0.47/3.81	67.9/3.99/54.2	0/1.63/0	0/0/0
method-3	98.2/41.2/95.1	4.72/0.71/20.3	81.7/1.25/82.0	2.58/0.40/4.88	0/0/0
method-4	98.6/39.8/95.3	9.15/0.56/38.6	83.2/1.21/82.7	0.26/0.38/0.53	0/0/-
method-5	94.7/31.2/96.1	37.4/6.19/18.8	43.9/1.48/67.4	0.52/0.72/0.56	0/0/-
**proposed work**					
method-1	**94.1/36.3/95.4**	19.2/1.1/41.7	87.2/0.1/97.9	**89.2/4.1/10.7**	**100/0/100**
method-2	84.8/23.6/96.7	34/8.5/14	**99.6/0.8/90.3**	**61.5/4.9/6.5**	0/0/0
method-3	70.8/20.3/96.6	**42.4/20.1/7.9**	**99.7/0.7/90.4**	**63.8/6.3/5.3**	0/0/0
method-4	91.2/38/95.1	22.6/2.1/30.2	81.5/0.2/97.2	**90.4/5.7/8**	**100/0/100**

**Table 29:** Performance comparison with recent literature (N, S, and V classes).

Literature	N	S	V
	SEN/FPR/PPV	SEN/FPR/PPV	SEN/FPR/PPV
[61]	95/27.9/96.5	29.6/3.1/26.4	85.1/3.01/66.3
[62]	98.4/-/95.4	29.5/-/38.4	70.8/-/85.1
[63]			
method on VCG	79.1/27.0/96.3	31.2/8.4/13.0	89.5/7.2/46.1
**proposed work**			
method-1	**97.4/33.9/96.1**	19.9/1.6/33	**93.3/0.8/88.8**
method-2	89.1/24.6/96.9	34.1/8.9/13.5	**99.6/1.3/84.2**
method-3	76.2/21.6/96.8	**41.8/20.8/7.5**	**99.8/1.4/83.6**
method-4	95.8/35.7/95.8	23.3/2.4/28.2	88.1/1.6/79.9
